# The Utility of Urodynamic Studies in Neuro-Urological Patients

**DOI:** 10.3390/biomedicines11041134

**Published:** 2023-04-09

**Authors:** Andry Perrin, Jacques Corcos

**Affiliations:** Department of Urology, Jewish General Hospital, McGill University, Montreal, QC H3T 1E2, Canada

**Keywords:** urodynamic study, video-urodynamic study, utility, clinical performance, neurogenic lower urinary tract dysfunction, upper urinary tract deterioration

## Abstract

Introduction: The utility of a clinical tool lies in its clinical performance evaluation and describes the relevance and usefulness of that tool in a medical setting. The utility of urodynamic and video-urodynamic studies in the management of specific urodynamic profiles in the diagnosis, treatment, and prognostic approach in neuro-urological patients is the focus of the current review. Methods: For this narrative review, a PubMed^®^ search was performed by cross-referencing the keywords “urodynamics”, “neurogenic bladder”, “utility”, “clinical utility” and “clinical performance” with various terms related to the management of neurogenic lower urinary tract dysfunction. Clinical practice guidelines and landmark reviews from the most renowned experts in the field were also used. Analysis: Assessment of the utility of urodynamic study was performed during the diagnostic, therapeutic and prognostic steps of the neuro-urological patients’ management. We focused on its clinical performance in the identification and evaluation of several unfavorable events, such as neurogenic detrusor overactivity, detrusor-sphincter dyssynergia, elevated detrusor leak point pressure and the presence of vesico-ureteral reflux, which may be indicators for a higher risk for the development of urological comorbidities. Conclusion: Despite the paucity of existing literature assessing the utility of urodynamic study—specifically video-urodynamic study—in neuro-urological patients, it does remain the gold standard to assess lower urinary tract function precisely in this patient category. With regard to its utility, it is associated with high clinical performance at every step of management. The feedback on possible unfavorable events allows for prognostic assessment and may lead us to question current recommendations.

## 1. Introduction

Patients presenting with neurogenic lower urinary tract dysfunction (NLUTD) can develop a variety of clinical presentations. Invasive urodynamic study (UDS) is essential to objectively assess the detrusor and bladder outlet function. Simultaneous fluoroscopic imaging of the urinary tract during the UDS (video-urodynamic study, VUDS) is very useful for correlating UDS real-time events with anatomical findings and is therefore the most suitable procedure for investigating NLUTD [1].

UDS plays a central role in the evaluation of NLUTD, as it allows the investigator to assess the semiology, to identify a mixed presentation and to predict the risk of urological comorbidities [2]. Early diagnosis and treatment are essential to predict the occurrence of lower (LUT) and upper urinary tract (UUT) deterioration. Furthermore, LUT symptoms (LUTS) may be an initial presentation of neurological disease [3]. After treatment, UDS enables an objective assessment of the evolution of key parameters [4]. The role of surveillance UDS is debated, and existing guidelines show little consensus. Its use in the follow-up of patients at higher risk of future complications, even in those with stable clinical presentation, is generally recommended [5,6].

The utility of a clinical tool lies in its clinical performance evaluation and describes the relevance and usefulness of that tool in a medical setting. Clinical performance is defined as “the ability of a device, resulting from any direct or indirect medical effects which stem from its technical or functional characteristics, including diagnostic characteristics, to achieve its intended purpose as claimed by the manufacturer, thereby leading to a clinical benefit for patients, when used as intended by the manufacturer” [7]. The International Continence Society summarized the clinical performance requirements for UDS equipment in a dedicated guideline [8].

The utility of UDS and VUDS in the management of specific urodynamic profiles in the diagnosis, treatment and prognostic approach in patients with NLUTD is the focus of the current review. The intended audiences are primarily neuro-urologists, general urologists, physical medicine and rehabilitation practitioners, general practitioners, and specialized neuro-urological nurses.

## 2. Methods

In this narrative review, we performed a literature search by cross-referencing the keywords “urodynamics”, “neurogenic bladder”, “utility”, “clinical utility” and “clinical performance” with various terms related to the management of NLUTD on PubMed^®^. The search had no limitation with regard to language of publication and no date restrictions were applied. Clinical practice guidelines and landmark reviews from the most renowned experts in the field were also used [2,3,9]. We selected papers if these evaluated the utility and/or clinical utility of using urodynamic studies performed with or without fluoroscopy (VUDS) in the management of neuro-urological patients, with or without NLUTD. We also selected those evaluating the performance of these tests to achieve their intended purpose. Finally, we took into account those assessing the risk versus benefit associated with the use of these tests.

## 3. Analysis

### 3.1. UDS Diagnostic Performance

The performance of a diagnostic tool is assessed by parameters such as sensitivity, specificity, and positive and negative predictive values. Despite the lack of literature evaluating these parameters, UDS is currently the only method for assessing dysfunction of LUT during both the filling and voiding phases; therefore, it is the gold standard for the evaluation of the patient with NLUTD [10]. The features it can identify that may be indicators for a higher risk for the development of urological comorbidities are neurogenic detrusor overactivity (NDO), detrusor-sphincter dyssynergia (DSD), low bladder compliance (<20 mL/cmH2O), elevated detrusor leak point pressure (DLPP) and the presence of vesico-ureteral reflux (VUR) [11,12,13,14,15].

#### 3.1.1. UDS and NDO

NDO may be identified during the filling cystometry, which is performed using body-warm saline at a physiological filling rate. Some studies report a sensitivity ranging between 45–72% when UDS was compared to clinical symptoms of urge urinary incontinence in non-neurogenic patients with an overactive bladder [16]. In one study, UDS diagnosis of detrusor overactivity (instability) was made if the filling cystometry demonstrated a rise in true detrusor pressure of >15 cmH2O. This diagnosis was also made if a true detrusor rise of more than 5 cmH2O was associated with urethral relaxation and incontinence. All terminology, methods and diagnostic criteria were used according to recommendations published by the International Continence Society. Volumes were not mentioned in the studies reviewed [17]. As filling cystometry is the only technique available to assess the patient’s filling function, it is essential to highlight pathological findings like NDO and low bladder compliance [3]. The amplitude and duration of the NDO may predict the risk of renal deterioration [18]. It has a critical value in patients with spinal cord injury (SCI) or advanced multiple sclerosis (MS), in which such findings could be silent. After spinal shock, SCI patients should undergo timely baseline UDS to optimize their management, as early treatment of NLUTD and prevention of NLUTD complications [19], as proposed by Birkhäuser et al. [20], may improve patients’ long-term urological quality of life.

#### 3.1.2. UDS and DSD

The voiding cystometry (or pressure flow study) reflects the coordination between the detrusor and urethra or pelvic floor during the voiding phase. As most types of obstruction in NLUTD are due to DSD on a non-relaxing urethra and bladder neck, UDS may identify such findings in the context of clinically unclear voiding difficulties and an initial pathological free flowmetry (FF) and post-voiding residual urine measurement (PVR) [21,22]. DSD is diagnosed during the voiding phase of the UDS using electromyography [23]. The diagnosis of DSD during electromyography (EMG) is poorly standardized. EMG findings in DSD have been classified into three types by Blaivas [24]:

1. Type 1 DSD presents a progressive increase in the external urinary sphincter (EUS) activity, with a peak at maximal detrusor contraction followed by a quick relaxation of the EUS as the detrusor pressure declines, allowing urination.
2. Type 2 DSD shows clonic contractions of the EUS intermittently during the detrusor contraction, provoking intermittency of the urinary stream.
3. Type 3 DSD is characterized by a continuous EUS contraction during the entire detrusor contraction, resulting in urinary obstruction or inability to urinate.

A simplified classification has been suggested by Weld et al. by dividing DSD into two groups, continuous versus intermittent [25]. Both classifications are currently used. VUDS is valuable for the diagnosis of DSD [26]. The role of urethral pressure profilometry in diagnosing DSD is controversial and currently considered to be investigational [27]. Kurzrock and Polse demonstrated that the presence of a DSD was correlated to UUT impairment with 87% sensitivity, 70% specificity, a positive predictive value of 79% and a negative predictive value of 81% in 90 children with spinal dysraphism. These UUT impairments included anatomical changes such as the development of hydronephrosis, vesico-ureteral reflux and cortical loss. The UDS follow-up showed a resolution of the UUT damage by clean intermittent self-catheterization in approximately one-third of the patients presenting with a DSD [28].

#### 3.1.3. UDS and DLPP

As DLPP increases, so does the risk of renal function deterioration due to the transmission of the bladder pressure to the kidneys [29,30]. A cutoff of >40 cmH2O has been historically cited as representing higher risk for renal function impairment, but this was based on a study in children with spina bifida and is not applicable to adult NLUTD [13]. Moreover, DLPP’s low sensitivity for accurately predicting the risk of UUT and/or bladder damage makes it a poor diagnostic tool [31]. In their study demonstrating the relationship between UDS findings and UUT damage, Kurzrock and Polse reported that the presence of a DLPP >40 cmH2O was associated with UUT deterioration with a 36% sensitivity, 93% specificity, a positive predictive value of 85% and a negative predictive value of 57%. The UDS follow-up showed a resolution of the UUT damage by clean intermittent self-catheterization in approximately 36% of these patients [28].

#### 3.1.4. VUDS Contribution

**Figure 1:**
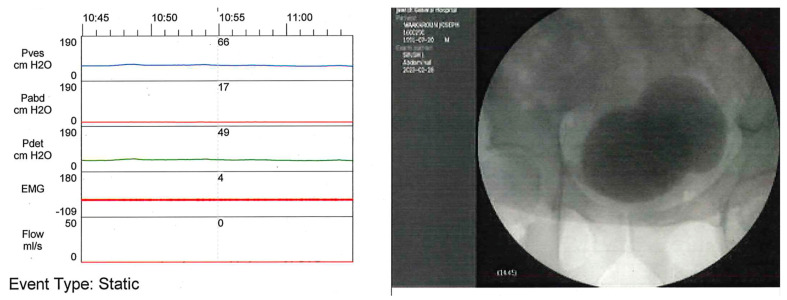
During the voiding phase of a VUDS, the fluoroscopic imaging allows us to identify a large bladder diverticulum, which results from a bladder neck obstruction (the urethra doesn’t fill with contrast). The detrusor contraction is normal (Green line) but the patient isn’t able to void (Orange line).

The additional correlation with imaging to the UDS allows evaluation of anatomical findings such as vesico-ureteral reflux (VUR), abnormal bladder morphology and bladder outlet obstruction during voiding (Figure 1) [32]. It had a clear advantage over conventional UDS when hydronephrosis was documented or when VUR was suspected or already known [33].

### 3.2. UDS Treatment Assessment Performance

There are three management goals in patients with NLUTD: to preserve the UUT function by maintaining a low storage pressure with proper bladder capacity and compliance and a low spontaneous voiding pressure; to minimize the occurrence of urinary tract infection (UTI); and to optimize continence. There are few data available supporting the impact of UDS on treatment outcomes in the neuro-urological patient population compared to “uncomplicated patients”, about whom most of the RCTs that assessed UDS’s clinical value have been published [10,34,35].

#### 3.2.1. UDS and NDO

Assessing the bladder volume and the height of the first uninhibited contraction (UIC) in the context of NDO is useful in evaluating the impact of a treatment.

Anticholinergic medications are the first line of treatment for NDO and may be combined with a beta-3-receptor agonist [36]. Treatment goals are to decrease the amplitude and duration of NDO, as well as to keep a compliant bladder with a suitable capacity. Improvements in UDS parameters have been highlighted in studies in adults with SCI or MS and children with myelodysplasia. However, not all patients responded to anticholinergic medication, and up to 8% of patients developed UUT changes, indicating the usefulness of surveillance [36,37,38,39]. Amarenco et al. showed that in a population of MS and SCI patients, after 4 weeks of treatment, solifenacin 5 mg PO DIE improved the maximum detrusor pressure (MDP) (−16.6 cmH2O) compared to placebo (+7.5 cmH2O). There wasn’t a dose-response relationship, as the MDP decrease was—10.5 cmH2O with solifenacin 10 mg PO DIE. It remained a significant improvement when compared to placebo. In the same study, oxybutynin 15 mg PO DIE showed an even better improvement (−24.3 cmH2O) [37].

UDS is strongly recommended before any invasive procedure, from intra-detrusor Botox^®^ (BTX) injections to bladder augmentation. In various studies evaluating the effectiveness of treatments or treatment modalities, UDS played a major role in the discussion of the current practice and recommendations [40,41,42].

New therapeutic strategies, such as transcutaneous tibial nerve stimulation to preempt the occurrence of NDO after acute SCI at an early stage, are the focus of the TASCI trial, in which UDS plays a major role in assessing the efficacy of treatment [20].

#### 3.2.2. UDS and DSD

In a substantial proportion of NLUTD patients with voiding dysfunction related to DSD, bladder drainage by a catheter is necessary. Self or caregiver-initiated intermittent catheterization (CIC) is the first-choice treatment. When CIC is not possible, patients can consider an indwelling urethral or suprapubic catheter. Difficulties or complications from catheterization may lead to more invasive procedures [23]. The role of UDS is more in the monitoring of the complications related to DSD than in evaluating the effectiveness of the treatment on the DSD itself.

#### 3.2.3. UDS and DLPP

DLPP superior to 40 cmH2O has been linked to long-term UUT damage. Kim et al. suggested that such pressure after sphincterotomy may indicate repeat surgery [43].

When elevated DLPP occurs only above a volume superior to the bladder capacity reported in the daily voiding pattern, it may not be physiologically relevant, but it helps to set the objectives of bladder drainage to maintain a low-pressure system.

Low DLPP is usually linked to urinary incontinence, and assessment of its severity may be of use for discussing treatment. Vainrib et al. advocated performing UDS in the case of myelomeningocele patients planned to undergo a bladder augmentation in case of urinary incontinence symptoms before surgery to rule out poor sphincteric function. If leakage is demonstrated, a concomitant continence-enhancing procedure should be considered, such as a urethral sling, artificial sphincter or complete bladder neck closure with a continent cutaneous urinary stoma [40].

DLPP measurement allows not only assessment of the indication and/or the success of a treatment but is also useful for evaluating the need for a concomitant or complementary intervention.

### 3.3. UDS Prognostic Evaluation Performance

Despite normal kidney function at the early stage of their neurological disease, neuro-urological patients are at risk for UUT damage with time. The clinical presentation of LUT dysfunction is unreliable for assessing UUT deterioration, particularly in the context of NLUTD [44].

Early and appropriate UDS-based management of patients with spinal cord injury was shown to prevent the long-term development of poor compliance requiring invasive and risky surgical treatment [19].

#### 3.3.1. UDS and NDO and DSD

NDO may impair quality of life when complicated by urinary incontinence, which not only leads to embarrassment, depression and social isolation but also may lead to skin decubiti and urethral erosions. Neuro-urological patients may also develop UUT damage and UTI when NDO is combined with obstructive patterns such as DSD.

NDO associated with DSD was the most related unfavorable VUDS findings reported by Kozomara et al. in up to 88% of 97 patients with traumatic or ischemic acute spinal cord injury in the first year after the incident. Early detection of the association between NDO and DSD allows for timely management of their potential complications [45]. Shin et al. have demonstrated that follow-up UDS can not only confirm improvement in bladder storage volume and pressure but also that it has been correlated to a better-estimated plasma flow on nuclear renogram [14].

#### 3.3.2. UDS and DLPP

High DLPP is known to represent a long-term threat for the UUT. In the same Swiss study previously mentioned, threatening maximum detrusor pressure during the storage phase, defined as superior to 40 cmH2O, has been observed in almost 40% of the patients followed up [1]. This widely accepted 40 cmH2O cut-off does not always reliably define safe storage pressure, as children—for example—may damage their UUT at lower thresholds [46,47]. The identification of any other concomitant unfavorable VUDS event is then critical.

#### 3.3.3. VUDS

VUDS is recommended by most authors as the study of choice for follow-up in patients with NLUTD, as it is the only test that may identify clinically silent anatomical variation with time, even though the evidence remains weak [48].

## 4. Discussion

### 4.1. Paucity of Data

Few studies assessed the utility of UDS and its impact on the management of NLUTD, and these were conducted mainly in small groups of consecutive adult and pediatric patients presenting NLUTD on SCI. Hence, conclusions must be drawn from studies conducted with different objectives, which affects the strength of our conclusion. Despite the paucity of data, we can observe that most patients required some form of intervention, ranging from a change in anticholinergic therapy or botulinum toxin injections to invasive surgery [1,28,49,50].

It is important to note that none of these articles dealt directly with the utility of UDS in the management of NLUTD. Therefore, the strength of the conclusions is limited by the fact that they have been drawn from studies with different objectives.

### 4.2. Medico-Economic Impact

As previously mentioned, UDS is the most accurate and objective method for evaluating the LUT function in the neuro-urological patient population. It is widely available in most developed countries. UDS is costly when taking into account the purchase of equipment and disposables, as well as the requirement for specialized staff. Early intervention based on urodynamic findings rather than initiating treatment only once complications of NLUTD are present could, however, spare resources and reduce overall costs [45,51].

### 4.3. Risk of UDS

UDS is an invasive procedure that causes discomfort and may provoke infection, bleeding and autonomic dysreflexia. The presence of asymptomatic bacteriuria does not seem to significantly influence the occurrence of UTI after the study or the UDS performance. Autonomic dysreflexia is a major concern, especially in patients with a lesion above the T6 spinal level. Blood pressure monitoring must be conducted in all quadriplegic patients and in those known for a previous history of autonomic dysreflexia undergoing UDS. In the latter, it is safer to give an alpha-blocker prophylaxis (such as terazosin 5 mg 30 min prior to UDS) or even general anesthesia. The indication for testing must be carefully weighed against the risk of complication with this life-threatening issue [52]. Radiation exposure must be considered in the follow-up strategy using repeated VUDS. In patients offered VUDS, following the recommendations of most guidelines, the cumulative effect of the radiation may be substantial [52]. Precautions should be exercised during the filling phase, especially in the context of an augmented bladder, bearing in mind the decreased bladder sensation and the risk of rupture of the weaker wall. Latex allergy should also be assessed to avoid atopic manifestations, ranging from minor reactions to anaphylaxis [52].

### 4.4. Surrogate to UDS

As UDS has associated costs, is time-consuming and relatively invasive, alternative clinical tools have been assessed to evaluate the need for UDS. For example, the correlation between the bladder wall thickness at various volumes and UDS findings showed that the bladder wall thickness was indeed useful for determining if a patient had risk factors for renal impairment. However, key clinical parameters such as NDO and incontinence could not be assessed and may require UDS evaluation regardless [53].

### 4.5. Consistency with International Guidelines

Existing guidelines recommend follow-up UDS evaluation, but there are few details regarding the incorporation of these recommendations into actual clinical practice (Table 1).

Adherence to clinical guidelines in practice varies considerably. Given the fact that UDS is the only method for objectively assessing any abnormalities of the LUT, this technique remains underutilized. Some studies have shown that a large proportion of patients presenting with NLUTD never underwent UDS investigation in the course of their management [56,57]. When referred, these patients’ initial management consisted of a focused history and clinical evaluation combined with a urinalysis and PVR measurement. Additional tests such as voiding diaries, pad-test, uroflowmetry, kidney imaging, cystoscopy and UDS were performed if clinically indicated at the initial assessment and follow-up.

Despite VUDS being advocated as the study of choice for the follow-up of neuro-urological patients, surveys carried out in Canada and the Netherlands showed that it is used only by a small number of urologists [58,59]. Recent studies in the UK showed an increase in adherence to the guidelines in recent years. A French survey observed that routine follow-ups including UDS were performed by 56% of urologists and 83% of physiatrists, most often annually [60,61,62].

## 5. Conclusions

Despite the paucity of existing literature assessing the utility of UDS in neuro-urological patients, UDS—specifically VUDS—does remain the gold standard for assessing LUT function precisely in this patient category.

With regard to its utility, UDS is associated with high clinical performance at every step of the NLUTD management. Its diagnostic performances allow for precise identification of risk factors predisposing to further urologic comorbidities and therefore tailoring of follow-up.

After treatment initiation, UDS provides relevant information about its efficacy, the need for adaptation, and additional interventions. Feedback on possible unfavorable events during NLUTD allows for prognostic assessment and may lead us to question current recommendations regarding follow-up. Periodic follow-up UDS should indeed be considered in neuro-urological patients who were offered an initial UDS assessment, particularly those who presented high-risk patterns or for whom complications were already apparent.

The performance of UDS lies in its unique ability to detect unfavorable patterns throughout diagnosis, treatment, and follow-up.

VUDS provides anatomical information with regard to LUT function available through no other exam, making it unavoidable in follow-up of NLUTD.

Unfavorable VUDS findings may reflect an impaired quality of life or lead to life-threatening complications, supporting the need for further research on the occurrence of these events to establish recommendations on VUDS follow-up in patients at higher risk of urological comorbidities and/or complications.

Eventually, bearing in mind a global trend toward better healthcare cost control, UDS remains an indispensable tool for early adaptation of management.

## Figures and Tables

**Table 1:** Recommendations of several associations on UDS in NLUTD patients.

Documents or Guidelines	Organization, Version	Recommendations	Level of Evidence/Grade of Recommendations	References
The AUA/SUFU Guideline on Adult Neurogenic Lower Urinary Tract Dysfunction: Diagnosis and Evaluation	American Urological Association, 2021	UDS, with or without fluoroscopy (VUDS), is recommended at initial evaluation of patients with unknown risk NLUTD and in the follow-up of those thought to be at moderate and high risk if change in signs and symptoms, new complications (autonomic dysreflexia, UTI, stones), UUT or renal function deterioration.	Moderate Recommendation; Evidence Level: Grade C	[2]
EAU Guidelines on Neuro-Urology	European Association of Urology, 2022	UDS investigation is the only method that can objectively assess the (dys-) function of the LUT.	2a	EAU Guidelines, 2022.
		VUDS is the optimum procedure for urodynamic investigation in neuro-urological disorders.	4	
		Perform urodynamic investigation as a mandatory baseline diagnostic intervention in high-risk patients at regular intervals.	Strong recommendation	
Urinary incontinence in neurological disease: assessment and management	National Institute for Health and Care Excellence, 2012	Do not offer UDS routinely to people at low risk of renal complications (most MS patients).		[54]
		Offer VUDS to people at high risk of renal complications (spina bifida, SCI or anorectal abnormalities).		
		Offer UDS before performing surgical treatments for NLUTD.		
Neurogenic Lower Urinary Tract Dysfunction: Clinical Management Recommendations	Fifth International Consultation on Incontinence 2013 (Published 2016)	UDS should selectively be employed to supplement clinical assessment in determining management in NLUTD		[55]
		UDS should be used to gauge potential impact on a renal function as a consequence of NLUTD.		
		Patients on CIC and bladder storage treatment often require long-term UDS and upper tract monitoring	A	
		Patients with stress incontinence in association with NLUTD require VUDS to evaluate both bladder and sphincter function	C	

## Abbreviations

BTX	Botox^®^
CIC	clean intermittent catheterization
DLPP	detrusor leak point pressure
DSD	detrusor-sphincter dyssynergia
EUS	external urinary sphincter
LUT	lower urinary tract
LUTS	lower urinary tract symptoms
MS	multiple sclerosis
NDO	neurogenic detrusor overactivity
NLUTD	neurogenic lower urinary tract dysfunction
PVR	post-voiding residual urine volume
SCI	spinal cord injury
UDS	urodynamic study
UUT	upper urinary tract
VUDS	video-urodynamic study
VUR	vesico-ureteral reflux

## References

[1] Nosseir M., Hinkel A., Pannek J. (2007). Clinical usefulness of urodynamic assessment for maintenance of bladder function in patients with spinal cord injury. Neurourol. Urodyn..

[2] Ginsberg D.A., Boone T.B., Cameron A.P., Gousse A., Kaufman M.R., Keays E., Kennelly M.J., Lemack G.E., Rovner E.S., Souter L.H. (2021). The AUA/SUFU Guideline on Adult Neurogenic Lower Urinary Tract Dysfunction: Diagnosis and Evaluation. J. Urol..

[3] Groen J. (2016). Summary of European Association of Urology (EAU) Guidelines on Neuro-Urology. Eur. Urol..

[4] Mangera A., Apostolidis A., Andersson K.E., Dasgupta P., Giannantoni A., Roehrborn C., Novara G., Chapple C. (2014). An updated systematic review and statistical comparison of standardised mean outcomes for the use of botulinum toxin in the management of lower urinary tract disorders. Eur. Urol..

[5] Winters J.C., Dmochowski R.R., Goldman H.B., Herndon C.D.A., Kobashi K.C., Kraus S.R., Lemack G.E., Nitti V.W., Rovner E.S., Wein A.J. (2012). Urodynamic studies in adults: AUA/SUFU guideline. J. Urol..

[6] Panicker J.N., de Sèze M., Fowler C.J. (2010). Rehabilitation in practice: Neurogenic lower urinary tract dysfunction and its management. Clin. Rehabil..

[7] Radley-Gardner O., Beale H., Zimmermann R. (2016). Fundamental Texts on European Private Law.

[8] Gammie A., Clarkson B., Constantinou C., Damaser M., Drinnan M., Geleijnse G., Griffiths D., Rosier P., Schäfer W., Van Mastrigt R. (2014). International Continence Society guidelines on urodynamic equipment performance. Neurourol. Urodyn..

[9] Drake M.J., Doumouchtsis S.K., Hashim H., Gammie A. (2018). Fundamentals of urodynamic practice, based on International Continence Society good urodynamic practices recommendations. Neurourol. Urodyn..

[10] Bodmer N.S., Wirth C., Birkhäuser V., Sartori A.M., Leitner L., Averbeck M.A., de Wachter S., Finazzi Agro E., Gammie A., Goldman H.B. (2022). Randomised Controlled Trials Assessing the Clinical Value of Urodynamic Studies: A Systematic Review and Meta-analysis. Eur. Urol. Open Sci..

[11] Welk B., Schneider M.P., Thavaseelan J., Traini L.R., Curt A., Kessler T.M. (2018). Early urological care of patients with spinal cord injury. World J. Urol..

[12] Musco S., Padilla-Fernández B., Del Popolo G., Bonifazi M., Blok B.F.M., Groen J., ’t Hoen L., Pannek J., Bonzon J., Kessler T.M. (2018). Value of urodynamic findings in predicting upper urinary tract damage in neuro-urological patients: A systematic review. Neurourol. Urodyn..

[13] Veenboer P.W., Bosch J.L.H.R., Rosier P.F.W.M., Dik P., van Asbeck F.W.A., de Jong T.P.V.M., de Kort L.M.O. (2014). Cross-sectional study of determinants of upper and lower urinary tract outcomes in adults with spinal dysraphism--new recommendations for urodynamic followup guidelines?. J. Urol..

[14] Shin J.C., Lee Y., Yang H., Kim D.H. (2014). Clinical significance of urodynamic study parameters in maintenance of renal function in spinal cord injury patients. Ann. Rehabil. Med..

[15] Ku J.H., Choi W.J., Lee K.Y., Jung T.Y., Lee J.K., Park W.H., Shim H.B. (2005). Complications of the upper urinary tract in patients with spinal cord injury: A long-term follow-up study. Urol. Res..

[16] Colli E., Artibani W., Goka J., Parazzini F., Wein A.J. (2003). Are urodynamic tests useful tools for the initial conservative management of non-neurogenic urinary incontinence? A review of the literature. Eur. Urol..

[17] De Muylder X., Claes H., Neven P., De Jaegher K. (1992). Usefulness of urodynamic investigations in female incontinence. Eur. J. Obstet. Gynecol. Reprod. Biol..

[18] Elmelund M., Klarskov N., Bagi P., Oturai P.S., Biering-Sørensen F. (2017). Renal deterioration after spinal cord injury is associated with length of detrusor contractions during cystometry-A study with a median of 41 years follow-up. Neurourol. Urodyn..

[19] Bywater M., Tornic J., Mehnert U., Kessler T.M. (2018). Detrusor Acontractility after Acute Spinal Cord Injury-Myth or Reality?. J. Urol..

[20] Birkhäuser V., Liechti M.D., Anderson C.E., Bachmann L.M., Baumann S., Baumberger M., Birder L.A., Botter S.M., Büeler S., Cruz C.D. (2020). TASCI-transcutaneous tibial nerve stimulation in patients with acute spinal cord injury to prevent neurogenic detrusor overactivity: Protocol for a nationwide, randomised, sham-controlled, double-blind clinical trial. BMJ Open.

[21] Al-Ali M., Haddad L. (1996). A 10 year review of the endoscopic treatment of 125 spinal cord injured patients with vesical outlet obstruction: Does bladder neck dyssynergia exist?. Int. Med. Soc. Paraplegia.

[22] Rossier A.B., Fam B.A. (1986). 5-microtransducer catheter in evaluation of neurogenic bladder function. Urology.

[23] Bacsu C.-D., Chan L., Tse V. (2012). Diagnosing detrusor sphincter dyssynergia in the neurological patient. BJU Int..

[24] Blaivas J.G., Sinha H.P., Zayed A.A., Labib K.B. (1981). Detrusor-external sphincter dyssynergia: A detailed electromyographic study. J. Urol..

[25] Weld K.J., Graney M.J., Dmochowski R.R. (2000). Clinical significance of detrusor sphincter dyssynergia type in patients with post-traumatic spinal cord injury. Urology.

[26] D’Ancona C., Haylen B., Oelke M., Abranches-Monteiro L., Arnold E., Goldman H., Hamid R., Homma Y., Marcelissen T., Rademakers K. (2019). The International Continence Society (ICS) report on the terminology for adult male lower urinary tract and pelvic floor symptoms and dysfunction. Neurourol. Urodyn..

[27] Lose G., Griffiths D., Hosker G., Kulseng-Hanssen S., Perucchini D., Schäfer W., Thind P., Versi E. (2002). Standardisation of urethral pressure measurement: Report from the Standardisation Sub-Committee of the International Continence Society. Neurourol. Urodyn..

[28] Kurzrock E.A., Polse S. (1998). Renal deterioration in myelodysplastic children: Urodynamic evaluation and clinical correlates. J. Urol..

[29] Gerridzen R.G., Thijssen A.M., Dehoux E. (1992). Risk factors for upper tract deterioration in chronic spinal cord injury patients. J. Urol..

[30] Ozkan B., Demirkesen O., Durak H., Uygun N., Ismailoglu V., Cetinel B. (2005). Which factors predict upper urinary tract deterioration in overactive neurogenic bladder dysfunction?. Urology.

[31] Linsenmeyer T.A., Bagaria S.P., Gendron B. (1998). The impact of urodynamic parameters on the upper tracts of spinal cord injured men who void reflexly. J. Spinal Cord Med..

[32] Wyndaele M., Rosier P.F.W.M. (2018). Basics of videourodynamics for adult patients with lower urinary tract dysfunction. Neurourol. Urodyn..

[33] Ginsberg D. (2013). The epidemiology and pathophysiology of neurogenic bladder. Am. J. Manag. Care.

[34] Majumdar A., Latthe P., Toozs-Hobson P. (2010). Urodynamics prior to treatment as an intervention: A pilot study. Neurourol. Urodyn..

[35] Kopp Kallner H., Elmér C., Altman D. (2019). Urodynamics as a Prognosticator of Mirabegron Treatment Outcomes. Gynecol. Obstet. Investig..

[36] Abrams P., Kelleher C., Staskin D., Rechberger T., Kay R., Martina R., Newgreen D., Paireddy A., van Maanen R., Ridder A. (2015). Combination treatment with mirabegron and solifenacin in patients with overactive bladder: Efficacy and safety results from a randomised, double-blind, dose-ranging, phase 2 study (Symphony). Eur. Urol..

[37] Amarenco G., Sutory M., Zachoval R., Agarwal M., Del Popolo G., Tretter R., Compion G., De Ridder D. (2017). Solifenacin is effective and well tolerated in patients with neurogenic detrusor overactivity: Results from the double-blind, randomized, active- and placebo-controlled SONIC urodynamic study. Neurourol. Urodyn..

[38] Hadiji N., Previnaire J.G., Benbouzid R., Robain G., Leblond C., Mieusset R., Enjalbert M., Soler J.M. (2014). Are oxybutynin and trospium efficacious in the treatment of detrusor overactivity in spinal cord injury patients?. Spinal Cord.

[39] Kasabian N.G., Bauer S.B., Dyro F.M., Colodny A.H., Mandell J., Retik A.B. (1992). The prophylactic value of clean intermittent catheterization and anticholinergic medication in newborns and infants with myelodysplasia at risk of developing urinary tract deterioration. Am. J. Dis. Child..

[40] Vainrib M., Reyblat P., Ginsberg D.A. (2013). Differences in urodynamic study variables in adult patients with neurogenic bladder and myelomeningocele before and after augmentation enterocystoplasty. Neurourol. Urodyn..

[41] Chen H., Xie K., Jiang C. (2021). A single-blind randomized control trial of trigonal versus nontrigonal Botulinum toxin-A injections for patients with urinary incontinence and poor bladder compliance secondary to spinal cord injury. J. Spinal Cord Med..

[42] Aharony S., Przydacz M., Van Ba O.L., Corcos J. (2020). Does asymptomatic bacteriuria increase the risk of adverse events or modify the efficacy of intradetrusor onabotulinumtoxinA injections?. Neurourol. Urodyn..

[43] Kim Y.H., Kattan M.W., Boone T.B. (1998). Bladder leak point pressure: The measure for sphincterotomy success in spinal cord injured patients with external detrusor-sphincter dyssynergia. J. Urol..

[44] Schöps T.-F., Schneider M.P., Steffen F., Ineichen B.V., Mehnert U., Kessler T.M. (2015). Neurogenic lower urinary tract dysfunction (NLUTD) in patients with spinal cord injury: Long-term urodynamic findings. BJU Int..

[45] Kozomara M., Birkhäuser V., Anderson C.E., Bywater M., Gross O., Kiss S., Knüpfer S.C., Koschorke M., Leitner L., Mehnert U. (2023). Neurogenic Lower Urinary Tract Dysfunction in the First Year After Spinal Cord Injury: A Descriptive Study of Urodynamic Findings. J. Urol..

[46] Tarcan T., Demirkesen O., Plata M., Castro-Diaz D. (2017). ICS teaching module: Detrusor leak point pressures in patients with relevant neurological abnormalities. Neurourol. Urodyn..

[47] Guerra L., Leonard M., Castagnetti M. (2014). Best practice in the assessment of bladder function in infants. Ther. Adv. Urol..

[48] Anding R., Rosier P., Smith P., Gammie A., Giarenis I., Rantell A., Thiruchelvam N., Arlandis S., Cardozo L. (2016). When should video be added to conventional urodynamics in adults and is it justified by the evidence? ICI-RS 2014. Neurourol. Urodyn..

[49] Linsenmeyer T.A., Linsenmeyer M.A. (2013). Impact of annual urodynamic evaluations on guiding bladder management in individuals with spinal cord injuries. J. Spinal Cord Med..

[50] Chao R., Mayo M.E. (1994). Long-term urodynamic follow up in pediatric spinal cord injury. Paraplegia.

[51] Abdel-Fattah M., Chapple C., Guerrero K., Dixon S., Cotterill N., Ward K., Hashim H., Monga A., Brown K., Drake M.J. (2021). Female Urgency, Trial of Urodynamics as Routine Evaluation (FUTURE study): A superiority randomised clinical trial to evaluate the effectiveness and cost-effectiveness of invasive urodynamic investigations in management of women with refractory overactive bladder symptoms. Trials.

[52] Sinha S. (2017). Follow-up urodynamics in patients with neurogenic bladder. Indian J. Urol..

[53] Pannek J., Bartel P., Göcking K., Frotzler A. (2013). Clinical usefulness of ultrasound assessment of detrusor wall thickness in patients with neurogenic lower urinary tract dysfunction due to spinal cord injury: Urodynamics made easy?. World J. Urol..

[54] Urinary Incontinence in Neurological Disease: Assessment and Management. https://www.nice.org.uk/guidance/cg148/resources/urinary-incontinence-in-neurological-disease-assessment-and-management-pdf-35109577553605.

[55] Drake M.J., Apostolidis A., Cocci A., Emmanuel A., Gajewski J.B., Harrison S.C.W., Heesakkers J.P.F.A., Lemack G.E., Madersbacher H., Panicker J.N. (2016). Neurogenic lower urinary tract dysfunction: Clinical management recommendations of the Neurologic Incontinence committee of the fifth International Consultation on Incontinence 2013. Neurourol. Urodyn..

[56] Welk B., Liu K., Shariff S.Z. (2016). The use of urologic investigations among patients with traumatic spinal cord injuries. Res. Rep. Urol..

[57] Liu J.S., Greiman A., Casey J.T., Mukherjee S., Kielb S.J. (2016). A snapshot of the adult spina bifida patient—High incidence of urologic procedures. Cent. Eur. J. Urol..

[58] Veenboer P.W., Ruud Bosch J.L.H., de Kort L.M.O. (2014). Assessment of bladder and kidney functioning in adult spina bifida patients by Dutch urologists: A survey. Neurourol. Urodyn..

[59] Blok B.F.M., Karsenty G., Corcos J. (2006). Urological surveillance and management of patients with neurogenic bladder: Results of a survey among practicing urologists in Canada. Can. J. Urol..

[60] Bycroft J., Hamid R., Bywater H., Patki P., Craggs M., Shah J. (2004). Variation in urological practice amongst spinal injuries units in the UK and Eire. Neurourol. Urodyn..

[61] Burki J.R., Omar I., Shah P.J.R., Hamid R. (2014). Long-term urological management in spinal injury units in the U.K. and Eire: A follow-up study. Spinal Cord.

[62] Denys P., Soler J.-M., Fatton B., Rischmann P., Yelnik A., Aegerter P., Saidji-Domingo N.-Y., Chartier-Kastler E. (2012). Highlighting differences in the management of neurogenic bladder existing between urologists and physiatrists: A survey conducted among 383 specialists. La Presse Médicale.

